# 

**Published:** 1895-05-18

**Authors:** 


**Notices of Births, Deaths, and Marriages** are inserted in this column at a charge of 2s. 6d. for an announcement not exceeding four lines.

**Notice to Advertisers and Subscribers.**

All Advertisements, Orders for Copies of The Hospital and Business Communications should be addressed to **The Manager, NOT TO THE EDITOR,** The Hospital, 428, Strand, London, W.C.

The Cost of Subscription, post free, is as follows:—Per week, 2½d.; per quarter, 2s. 8d.; per half-year, 5s. 3d.; per annum, 10s. 6d. Foreign:—Per Annum, 15s. 2d.; payable, in all cases, in advance.

### NOTICE TO CORRESPONDENTS.

All MS., Letters, Books for Review, and other matters intended for the Editor should be addressed The Editor, The Lodge, Porchester Square, London, W.

The Editor cannot undertake to return rejected MS., even when accompanied by stamped directed envelope.

**"Burdett's Hospital Annual," 1895.**

Ready Immediately.

Sixth year of publication. About 1000 pp. Scarlet cloth, gilt lettered. Price 5s. A most complete guide to British, Colonial, Indian, and American Hospitals, Dispensaries, Nursing and Convalescent Institutions, and Asylums. A few copies of the "Annual" for 1894 may still be obtained on application to the Publishers, The Scientific Press (Limited), 428, Strand, London, W.C.

**NOTICE.—Vol. XVII. of "The Hospital" (October, 1894, to April, 1895), in handsome cloth binding, is now on sale at the Office of this Journal. Price 6s. Cases for binding the Numbers contained in this Volume, 1s. 6d. Index to Vol. XVII., 2½d., post free.**

### SCRAPS AND GLEANINGS.

Mr. Crosby Lockwood, of Highbury New Park, has bequeathed £50 to the Metropolitan Hospital Sunday Fund.

Monsieur Faure continues to show great interest in charities, and visits hospitals wherever his official duties lead him.

St. Mary's Hospital, Paddington, has just received, through Mr. G. Herrey, a further donation of £300 from Baron de Hirsch.

A Red Cross hospital, fully equipped, has been sent to Africa for the use of a Native Army, the whole expense being borne by the society.

If the Duchess of Sutherland is successful in her excellent efforts Sutherlandshire will be provided with a nurse for each of its villages.

The extern department of the Belfast Ophthalmic Hospital has undergone structural alterations, giving increased facility for the treatment of the patients.

Lord Knutsford has been appointed by the Prince of Wales to succeed Lord Sandhurst as director and chairman of the St. John Ambulance Association.

We learn from America that a Bill has been introduced regulating the fees of the medical men in the Illinois Legislation. One clause in the Bill puts the maximum surgical fee at 100 dollars.

Sir Henry Watson, chairman to the board of the Children's Hospital Sheffield, has signified his willingness to assist in promoting a fund for the erection of a mortuary to the hospital, which addition is much needed.

We are glad to see that the income of the Nottingham General Hospital is steadily increasing. The amount received from the Hospital Saturday collections, for instance, being £250 more than in the preceding year.

The North Ormesby Cottage Hospital authorities have arranged to apply the legacy of £1,000 from the late Mr. Hymers, of Stokesley, for the purpose of making some structural alterations and additions to the hospital.

The fifty-first annual meeting of the Church of England Scripture Readers' Association was held on Wednesday, May 15th, at Prince's Hall, Piccadilly. The chair was taken by the Right Rev. the Bishop of Stepney.

The ward in the new wing of the West Ham Hospital named after Mrs. Passmore Edwards will be furnished for the institution by her husband, who bore the cost of the erection of this extension himself. In aid of the funds of the hospital the Strolling Players' Amateur Dramatic Society gave a performance in the Town Hall.

A scheme for a National Bazaar is being inaugurated by the Society for the Prevention of Cruelty to Children; the branches in the country holding the bazaar in October or November, the metropolitan branch being in June. The society are forwarding the scheme with the object of establishing a "Reserve Fund." H.M. the Queen, has graciously signified her consent to become a patroness.

The Saddlers' Company have contributed 100 guineas to the St, Thomas's Hospital Appeal Fund, and have also sent 50 guineas to each of the following charities: Royal London Ophthalmic Hospital, Royal Eye Hospital, North-Eastern Hospital for Children, Dental Hospital, City of London Chest Hospital, All Saints' Convalescent Hospital, Eastbourne, Royal Maternity Charity, and the Surgical Aid Society. £100 has also been sent to the Gordon Boys' Home.

Liverpool has been presented, through an unknown donor, with a park of 108 acres.

Donations to the extent of £2,500 are announced to have been received after the annual dinner in connection with King's College Hospital.

The number of patients who have passed through the Derbyshire Royal Infirmary Convalescent Home at Holbrooke was 127 during last year.

The financial condition of the Royal Hospital for Incurables, at Dublin, is under serious consideration, there being over £2,000 due to the bank.

The twenty-seventh annual report of the Felixstowe Convalescent Home states that 386 patients were admitted during the year. The finances of the institution are in a flourishing condition.

The cost of the reconstruction of Torbay Hospital was larger than had at first been expected, the restoration up to date being £10,000. A sum of £250 towards this was subscribed for refurnishing, in addition to which special gifts have been made to the hospital.

At the Glasgow Hospital for Skin Diseases 1,028 cases have been admitted into the wards during the year. A still greater improvement in the financial contributions from working men's subscriptions has been shown.

The discussion as to a suitable site for the Newcastle Infirmary continues to be undecided, although it is agreed on all hands that further accommodation for the efficient carrying on of the work of the infirmary is absolutely necessary.

The funds of the Chichester Infirmary are at a low ebb, and the attention of the West Sussex public has been called to the needs of so important an institution, indeed, unless special donations are forthcoming the necessity for selling part of the investments cannot be averted.

It was pointed out by the promoters of the Hospital Sunday collection at Coventry that unless further financial support was given towards the funds of the institution there would be a grave necessity on the part of the committee to take the retrograde step of closing some of the wards, to which fact we have already referred.

The Sheffield Hospital Saturday Committee, in their annual appeal, point out that a special effort should be made this year to swell the funds of the institutions which benefit by the fund. The principal reason is that the annual reports of the four hospitals among which the money is divided show an aggregate deficiency of £4,662.

A record of good work is presented in this the twenty-fifth annual report of the Wimbledon Cottage Hospital. The receipts, though they have fallen short of those in 1893 have sufficed to cover the ordinary expenditure during the twelve months under review. The extraordinary expenditure has been defrayed out of the balance in hand at the commencement of the year.

The work accomplished by the City of London Truss Society is too well known to need comment. The numbers of sufferers who benefit by its help increase yearly, and there is of course the consequent need for proportionate augmentation of funds. The festival dinner is arranged to take place on June 14th, when the Bishop of Derry will preside, and we cordially hope that a large subscription list will result. The society is one which deserves and should have no difficulty in obtaining the required support from its friends and well-wishers.

### THE STUDENT OF MEDICINE.

### APPOINTMENTS.

Dr. G. R. Adcock, appointed District Medical Officer to the Hoxne Union. R. T. Bakewell, M.B., M.R.C.S., appointed Anæsthetist to the Hospital for Sick Children, Great Ormond Street. J. S. Boden, M.R.C.S., L.R.C.P., appointed House Accoucheur to King's College Hospital. N. T. Bond, M.B., C.M.Edin., appointed Medical Officer for the No. 5 District of the Liskeard Union. A. Bousfield, B.A.Cantab, B.Sc.Lond., M.R.C.S., L.R.C.P., appointed Assistant House-Physician to King's College Hospital. H. A. Burridge, M.R.C.S., L.R.C.P., appointed Assistant House Accoucheur to King's College Hospital. R. Crawford, M.A., M.B., B.Ch.Oxon, M.R.C.P.Lond., appointed House Physician to King's College Hospital. Dr. Davidson, appointed Medical Officer for the Eaton District of the Macclesfield Union. F. Edmunds, M.R.C.S.Eng., L.R.C.P.Lond., appointed Medical Officer for the Chesterfield District and the Workhouse of the Chesterfield Union. E. C. Edwards, M.B., C.M.Edin., appointed Senior House Surgeon to the East Suffolk Hospital, Ipswich. H. C. Ensor, M.R.C.S.Eng., L.S.A., appointed Assistant Ophthalmic Surgeon to the Cardiff Infirmary. E. F. Grün, L.R.C.P.Lond., M.R.C.S.Eng., appointed Medical Officer for the No. 2 (Southwick) District of the Steyning Union. A. Howie, M.B. and C.M., appointed Medical Officer for the Alberbury District of the Atcham Union. J. S. Jameson, M.D., B.S., B.A.O.Dubl., appointed Medical Officer and Public Vaccinator to the Unions of Wix, Ardleigh, and Great Bromley. A. E. Kempthorne, M.R.C.S.Eng., L.S.A., appointed Medical Officer for the Second District of the Parish of St. Matthew, Bethnal Green. Dr. G. H. Oliver, appointed Assistant Surgeon to the Bradford Eye and Ear Hospital. R. S. Olver, M.R.C.S., L.R.C.P., appointed House Surgeon to King's College Hospital. D. R. Paterson, M.D.Edin., M.R.C.P.Lond., appointed Assistant Physician to the Cardiff Infirmary. F. S. Penny, M.R.C.S., L.R.C.P., appointed House Surgeon to King's College Hospital. T. E. Rice, L.S.A.Lond., appointed House Surgeon to King's College Hospital. A. W. Sheen, M.D.Lond., F.R.C.S.Eng., appointed Assistant Surgeon to the Cardiff Infirmary. Amy Sheppard, M.B Lond., appointed Assistant Ophthalmic Surgeon to the New Hospital for Women, Euston Road. G. U. Smith, M.R.C.S., L.R.C.P., appointed Ophthalmic Clinical Assistant to King's College Hospital. J. W. Taylor, M.R.C.S.Eng., L.R.C.P.Lond., appointed Medical Officer of Health for the Methley Urban District Council. J. L. Thomas, F.R.C.S.Eng., L.R.C.P.Lond., appointed Assistant Surgeon to the Cardiff Infirmary. E. J. Treasurywálá, M.D., L.R.C P. & S., appointed Assistant Electrician at St. Bartholomew's Hospital.

The annual dinner of old students of King's College, London, will be held at the Holborn Restaurant on Monday, June 24th, with the Right Rev. the Lord Bishop of London, D.D., in the chair.

### VACANCIES.

The following vacancies are announced this week, the amount of salary in each case being given after the name of the institution:

### Resident Medical Officers.

Asylum for Idiots, Earlswood, Redhill, Surrey.—Assistant Medical Officer. Salary £150 per annum, with board and residence. Applications, marked "Assistant Medical Officer," to the Secretary, 36, King William Street, London Bridge, E C., by May 23rd.

Blackburn and East Lancashire Infirmary.—Junior House Surgeon, will be required to enter into an agreement not to commence private practice within the Borough of Blackburn for a period of three years. Salary £50 per annum, with board, washing, lodging, &c. Applications and testimonials to N. A. Smith, Secretary, by May 23rd.

General Hospital, Birmingham.—Assistant House Surgeon. Must possess surgical qualification and be registered. Appointment for six months. No salary, but residence, board, and washing provided. Applications, with certificate of registration and copies of testimonials, to H. J. Collins, House Governor, by June 1st.

Halifax Infirmary and Dispensary.—House Surgeon. Must be unmarried, doubly qualified, and registered. Salary £80 per annum, advancing £10 per annum up to £100, with residence, board, and washing. Applications and testimonials to Oates Webster, Secretary, by May 22nd.

Kensington Workhouse Infirmary.—Second Assistant Resident Medical Officer. Must be between 21 and 30 years of age. Must possess both a medical and surgical qualification. Salary £80 per annum, with apartments, board, and washing. Applications and testimonials to J. H. Rutherglen, Clerk to the Guardians, by May 25th.

Western General Dispensary, Marylebone Road.—Junior House Surgeon. Must be duly registered and unmarried. Salary £50 per annum, with rooms and board. Applications and testimonials to the Honorary Secretary by May 24th.

Westminster General Dispensary, 9, Gerrard Street, Soho, W.—Honorary Surgeon. Applications and testimonials to J. J. Johnson, Secretary, by May 20th.

Worcester General Infirmary, Worcester.—Assistant House Surgeon and Dispenser, fully qualified: unmarried. Salary £70 per annum, with board, residence, and washing. Office tenable for not more than two years. Applications and testimonials to William Stallard, Secretary, Worcester Chambers, Pierpoint Street, Worcester, by May 18th.

### Non-Resident Medical Officers.

Dental Hospital of London, Leicester Square.—Two Dental Surgeons. Candidates must be Licentiates of Dental Surgery of one of the licensing bodies recognised by the General Medical Council. Applications and testimonials to J. Francis Pink, Secretary, by June 10th.

Great Northern Central Hospital, Holloway Road, N.—Pathologist and Registrar. Appointment for one year. Honorarium at the rate of fifty guineas per annum. Applications to the Secretary by May 27th.

Royal Westminster Ophthalmic Hospital, King William Street, West Strand.—Clinical Assistants. Appointments for six months. Must be duly qualified, and preference will be given to those who have had previous experience of ophthalmic practice. Applications and testimonials to T. Beattie-Campbell, Secretary, by June 1st.

St. Thomas's Hospital Medical School.—Lecturer on Physiology. Applications to the Dean by May 25th.

